# Thiamine use is associated with better outcomes for traumatic brain injury patients

**DOI:** 10.3389/fnut.2024.1362817

**Published:** 2024-07-05

**Authors:** Ruoran Wang, Yunhui Zeng, Jianguo Xu, Min He

**Affiliations:** ^1^Department of Neurosurgery, West China Hospital, Sichuan University, Chengdu, Sichuan, China; ^2^Department of Critical Care Medicine, West China Hospital, Sichuan University, Chengdu, Sichuan, China

**Keywords:** traumatic brain injury, thiamine, mortality, vitamin B1, nutrition

## Abstract

**Background:**

Traumatic brain injury (TBI) is a global health concern that often leads to poor prognosis. We designed this study to explore whether thiamine use is associated with a better prognosis of TBI.

**Methods:**

TBI patients selected from the Medical Information Mart for Intensive Care-III database were included in the study. Univariate and multivariate Cox regression analyses were performed to examine the relationship between thiamine use and mortality in TBI patients. Propensity score matching (PSM) was utilized to generate balanced cohorts of the non-thiamine use group and the thiamine use group. Subgroup analysis was performed in the cohort after PSM to verify the association between thiamine use and mortality in TBI patients across different stratifications.

**Results:**

The incidence of thiamine use in TBI was 18.3%. The thiamine use group had a lower 30-day mortality rate (*p* < 0.001), a longer length of ICU stay (*p* < 0.001), and a longer length of hospital stay (*p* < 0.001) than the non-thiamine use group, both in the primary cohort before PSM and the cohort after PSM. A multivariate Cox regression analysis confirmed that thiamine use was independently associated with mortality (OR = 0.454, *p* < 0.001) after adjusting for confounding effects. In the cohort after PSM, the subgroup analysis showed that thiamine use is associated with lower mortality in TBI patients with a Glasgow Coma Scale (GCS) score of < 13, but it is not associated with mortality in TBI patients whose GCS score is ≥13.

**Conclusion:**

Thiamine supplementation is effective in improving the outcome of TBI, except in cases of mild TBI. The optimal thiamine supplementation strategy for TBI is worthwhile to be explored in future studies.

## 1 Introduction

Traumatic brain injury (TBI) is a public health concern with high morbidity and mortality rates. The estimated incidence of TBI is 69 million per year globally (1). Although many advances have been achieved to improve the prognosis of TBI, including temperature management, hypertonic saline, and multimodal monitoring (2, 3), research studies exploring the effectiveness of pharmacological interventions aimed at TBI, including nutritional supplementation, have never been interrupted (4, 5). Poor nutritional status is commonly observed in TBI patients and is associated with worse prognosis in these patients (6–8). The poor nutritional status of TBI patients may be attributable to multiple factors, including increased metabolic demand due to systemic inflammation, massive bleeding, and reduced absorption due to dysphagia and gastrointestinal dysfunction. Nutritional supplements including vitamins and trace elements are very important in improving the prognosis of critically ill patients, including those with TBI (9–11). Previous studies have investigated the influence of several nutrient supplements on the outcome of TBI animal models, including omega-3, zinc, vitamin D, vitamin E, and glutamine (12–16).

As an important member of B vitamins, thiamine participates in the formation of coenzymes for many important enzymes responsible for the carbohydrate metabolism, including pyruvate dehydrogenase, α-ketoglutarate dehydrogenase, and transketolase. The pyruvate dehydrogenase and α-ketoglutarate dehydrogenase catalyze the ATP production in the mitochondrion, while the transketolase promotes the pentose-phosphate cycle to produce ribose-5-phosphate and nicotinamide adenine dinucleotide phosphate, which play an important role in the biosynthesis of nucleotides, fatty acids, cholesterol, and steroid hormones (17, 18). Thiamine deficiency can disturb the carbohydrate metabolism with decreased energy production and the accumulation of lactate. Some studies have explored the effect of thiamine supplementation on the prognosis of several kinds of patients (19–22). While no clinical study has yet explored the effect of thiamine supplementation on the prognosis of TBI patients, we designed this study to verify the effect of thiamine supplementation on mortality in TBI patients and identify those who could benefit from the supplementation.

## 2 Materials and methods

### 2.1 Patients

**Figure 1:**
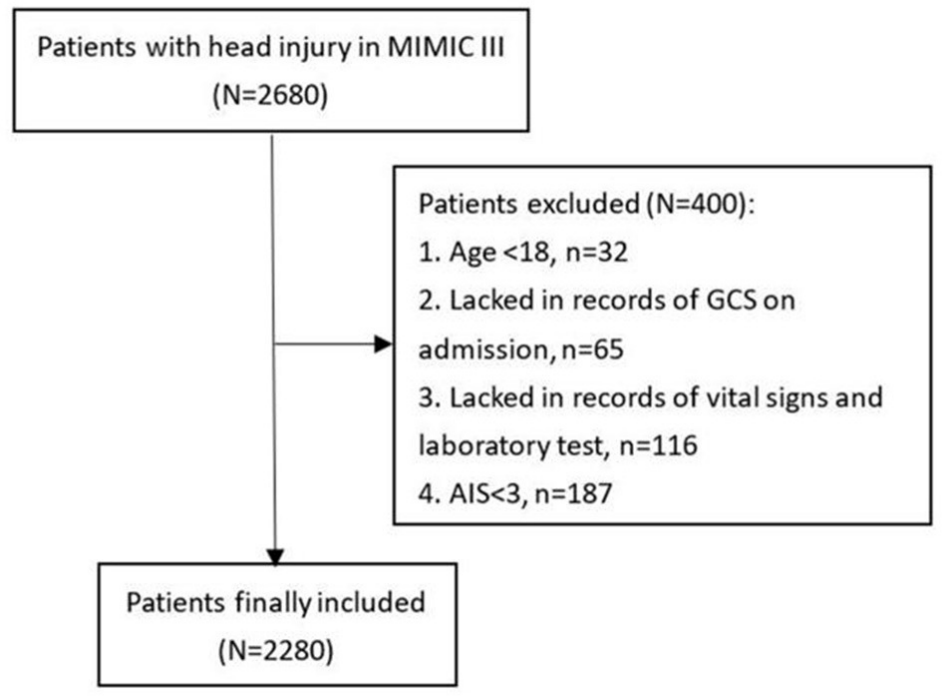
Flowchart of patient inclusion.

TBI patients selected from the Medical Information Mart for Intensive Care-III (MIMIC-III) database were included in this study. The MIMIC-III is a freely available database that compiles clinical information of patients hospitalized in the intensive care units of the Beth Israel Deaconess Medical Center (BIDMC) (Boston, MA) between 2001 and 2012. This database received ethical approval from the institutional review boards of the Massachusetts Institute of Technology (Cambridge, MA) and BIDMC. All patients recorded in this database were anonymized and de-identified to protect their privacy. The diagnosis of TBI was confirmed based on the following ICD-9 codes: 80000–80199; 80300–80499; and 8500–85419. Eligible TBI patients were excluded if they met the following criteria: (1) age < 18 years; (2) missing records of the Glasgow Coma Scale (GCS) score on admission; (3) missing records of vital signs on admission and laboratory tests; and (4) an Abbreviated Injury Scale (AIS) score for the head of < 3. A total of 2,280 patients were finally included in the study (Figure 1).

### 2.2 Data collection

Patients' age, gender, and comorbidities, including diabetes mellitus, hypertension, coronary heart disease, history of myocardial infarction, cerebral vascular disease, liver disease, chronic renal disease, and cancer, were collected. Upon admission, vital signs including systolic blood pressure, diastolic blood pressure, and heart rate were recorded. Additionally, the initial GCS and Injury Severity Scale (ISS) scores were collected to evaluate the severity of injuries. Laboratory tests for white blood cell (WBC) count, platelet count, hemoglobin level, and glucose level were analyzed from the first blood sample within 24 h of admission. Intracranial injury types, including epidural hematoma, subdural hematoma, subarachnoid hemorrhage, and intraparenchymal hemorrhage, were identified. The coagulopathy was confirmed based on the following criteria: activated partial thrombin time (APTT)>40s, or/and international normalized ratio (INR)>1.2, or/and platelet < 120 × 10^9^/L (23, 24). Treatments including red blood cell (RBC) transfusion during the first day, platelet transfusion during the first day, vasopressor use during the first day, mechanical ventilation, and neurosurgery were recorded. The primary outcome of this study was the 30-day mortality rate. The length of ICU stay and length of hospital stay were compared between the non-thiamine group and the thiamine group.

### 2.3 Statistical analysis

The Kolmogorov–Smirnov test was utilized to identify the normality of variables. Variables with normal or non-normal distributions were presented as mean ± standard deviation and median (interquartile range), respectively. Student's *t*-test and the Mann–Whitney *U-*test were utilized to compare the difference between the two groups of variables with normal or non-normal distributions, respectively. Categorical variables were presented as counts (percentage). The Chi-squared test or Fisher exact test was applied to compare the difference between the two groups of categorical variables. A univariate Cox regression analysis was performed to discover the potential risk factors of mortality in TBI patients. The association between thiamine use and mortality in TBI patients was confirmed using the multivariate Cox regression analysis after adjusting for risk factors discovered in the univariate Cox regression. To reduce the impact of bias and confounding variables on the association, PSM was performed to generate baseline balanced cohorts with a matching ratio of 1:1. Differences in survival rates between the thiamine use group and the non-thiamine use group were verified by the Kaplan–Meier survival analysis in the primary cohort before PSM and in the cohort after PSM. The association between thiamine use and mortality was confirmed by the univariate Cox regression analysis in the cohort after PSM. Subgroup analysis was also performed in the cohort after PSM, stratified by age, gender, GCS score, and ISS score. The relationship between thiamine dose and mortality was analyzed using the Restricted Cubic Spline (RCS) curve among TBI patients receiving thiamine in the cohort after PSM.

A two-sided *p* < 0.05 was considered to be statistically significant. R (version 3.6.1; R Foundation) software was used to perform statistical analyses and generate figures.

## 3 Results

### 3.1 Baseline characteristics of TBI patients

In this study, 18.3% of TBI patients used thiamine (Table 1). In the primary cohort before PSM, the thiamine use group had a lower average age (*p* < 0.001), a higher percentage of male individuals (*p* < 0.001), and a lower incidence of complicated diabetes (*p* = 0.010), hyperlipidemia (*p* = 0.044), coronary heart disease (*p* < 0.001), chronic renal disease (*p* = 0.017), and cancer (*p* = 0.017). In the thiamine use group, both the initial diastolic blood pressure (*p* < 0.001) and heart rate (*p* < 0.001) were significantly higher, while the GCS (*p* < 0.001) score was significantly lower. The results of laboratory tests indicated that, compared to the non-thiamine use group, the thiamine use group had lower WBC count (*p* = 0.024), platelet count (*p* = 0.030), and glucose level (*p* < 0.001) but higher hemoglobin level (*p* < 0.001). Additionally, RBC transfusion (*p* = 0.020) and vasopressor use (*p* = 0.039) during the first day were more prevalent in the non-thiamine use group. Finally, compared with the non-thiamine use group, the thiamine use group had a higher incidence of mechanical ventilation use (*p* < 0.001), lower 30-day mortality (*p* < 0.001), a longer length of stay in the ICU (*p* < 0.001), and a longer length of hospital stay (*p* < 0.001). A total of 804 TBI patients were included and compared after PSM with the match ratio of 1:1. In the cohort after PSM, the thiamine use group still had a lower 30-day mortality (*p* < 0.001) rate, a longer length of ICU stay (*p* < 0.001), and a longer length of hospital stay (*p* < 0.001) compared to the non-thiamine use group.

**Table 1:** Baseline characteristics of TBI patients cohort before PSM and after PSM.

	**Cohorts before PSM**				**Cohorts after PSM**			
	**Overall patients (*****n** =* **2,280)**	**Non-thiamine group (*****n** =* **1,863, 81.7%)**	**Thiamine group (*****n** =* **417, 18.3%)**	**p**	**Overall patients (*****n** =* **804)**	**Non-thiamine group (*****n** =* **402)**	**Thiamine group (*****n** =* **402)**	**p**
Age (year)	64.9 (43.7–81.0)	70.2 (44.2–83.0)	52.7 (43.0–63.2)	**< 0.001**	52.9 (37.0–68.3)	52.9 (28.6–74.3)	52.8 (43.0–63.7)	0.665
Male gender (%)	1400 (61.4%)	1070 (57.4%)	330 (79.1%)	**< 0.001**	637 (79.2%)	322 (80.1%)	315 (78.4%)	0.543
**Comorbidities**								
Diabetes (%)	351 (15.4%)	304 (16.3%)	47 (11.3%)	**0.010**	92 (11.4%)	45 (11.2%)	47 (11.7%)	0.825
Hypertension (%)	844 (37.0%)	703 (37.7%)	141 (33.8%)	0.134	273 (34.0%)	136 (33.8%)	137 (34.1%)	0.941
Hyperlipidemia (%)	298 (13.1%)	256 (13.7%)	42 (10.1%)	**0.044**	86 (10.7%)	44 (10.9%)	42 (10.4%)	0.819
Coronary heart disease (%)	293 (12.9%)	260 (14.0%)	33 (7.9%)	**< 0.001**	64 (8.0%)	31 (7.7%)	33 (8.2%)	0.794
History of myocardial infarction (%)	83 (3.6%)	71 (3.8%)	12 (2.9%)	0.358	24 (3.0%)	12 (3.0%)	12 (3.0%)	1.000
Cerebral vascular disease (%)	41 (1.8%)	35 (1.9%)	6 (1.4%)	0.541	11 (1.4%)	5 (1.2%)	6 (1.5%)	0.761
Liver disease (%)	94 (4.1%)	51 (2.7%)	43 (10.3%)	**< 0.001**	61 (7.6%)	30 (7.5%)	31 (7.7%)	0.894
Chronic renal disease (%)	153 (6.7%)	136 (7.3%)	17 (4.1%)	**0.017**	26 (3.2%)	10 (2.5%)	16 (4.0%)	0.232
Cancer (%)	238 (10.4%)	208 (11.2%)	30 (7.2%)	**0.017**	57 (7.1%)	27 (6.7%)	30 (7.5%)	0.680
**Vital signs on admission**								
Systolic blood pressure (mmHg)	132 (117–147)	132 (117–147)	131 (115–147)	0.273	131 (116–145)	130 (117–143)	131 (114–147)	0.482
Diastolic blood pressure (mmHg)	67 (56–77)	66 (56–76)	70 (59–82)	**< 0.001**	69 (59–81)	69 (60–79)	69 (59–82)	0.607
Heart rate (min^−1^)	83 (72–96)	82 (71–95)	89 (77–100)	**< 0.001**	87 (76–100)	87 (75–100)	88 (77–100)	0.503
GCS	12 (6–15)	13 (6–15)	10 (6–14)	**< 0.001**	10 (6–15)	9 (6–15)	10 (6–14)	0.742
ISS	16 (16–25)	16 (16–25)	16 (14–25)	0.368	16 (16–25)	16 (16–25)	16 (14–25)	0.201
**Laboratory tests**								
WBC (10^9^/L)	11.60 (8.40–15.70)	11.70 (8.70–15.70)	11.20 (7.80–15.70)	**0.024**	11.60 (8.30–15.80)	11.80 (8.80–15.80)	11.30 (8.00–15.70)	0.160
Platelet (10^9^/L)	230 (183–285)	231 (185–285)	226 (165–281)	**0.030**	228 (182–283)	228 (187–283)	227 (170–284)	0.330
Hemoglobin (g/dL)	12.8 (11.4–14.1)	12.7 (11.3–14.0)	13.1 (11.8–14.5)	**< 0.001**	13.2 (11.9–14.6)	13.3 (12.1–14.6)	13.1 (11.8–14.5)	0.210
Glucose (mg/dL)	132 (110–165)	134 (111–167)	124 (105–150)	**< 0.001**	123 (105–150)	123 (106–150)	124 (105–151)	0.697
**Intracranial injury locations**								
Epidural hematoma (%)	543 (23.8%)	434 (23.3%)	109 (26.1%)	0.218	208 (25.9%)	104 (25.9%)	104 (25.9%)	1.000
Subdural hematoma (%)	1319 (57.9%)	1097 (58.9%)	222 (53.2%)	**0.035**	423 (52.6%)	211 (52.5%)	212 (52.7%)	0.944
Subarachnoid hemorrhage (%)	958 (42.0%)	779 (41.8%)	179 (42.9%)	0.678	341 (42.4%)	169 (42.0%)	172 (42.8%)	0.830
Intraparenchymal hemorrhage (%)	447 (19.6%)	348 (18.7%)	99 (23.7%)	**0.019**	183 (22.761%)	88 (21.9%)	95 (23.6%)	0.556
Coagulopathy (%)	743 (32.6%)	611 (32.8%)	132 (31.7%)	0.653	237 (29.478%)	113 (28.1%)	124 (30.8%)	0.395
RBC transfusion during the first day (%)	178 (7.8%)	157 (8.4%)	21 (5.0%)	**0.020**	39 (4.9%)	19 (4.7%)	20 (5.0%)	0.870
Platelet transfusion during the first day (%)	223 (9.8%)	187 (10.0%)	36 (8.6%)	0.383	71 (8.8%)	38 (9.5%)	33 (8.2%)	0.534
Vasopressor during the first day (%)	150 (6.6%)	132 (7.1%)	18 (4.3%)	**0.039**	32 (4.0%)	14 (3.5%)	18 (4.5%)	0.471
Thiamine dose (mg)	0	0	200 (100–300)	**< 0.001**	100 (0–200)	0	200 (100–300)	**< 0.001**
Mechanical ventilation (%)	1034 (45.4%)	789 (42.4%)	245 (58.8%)	**< 0.001**	456 (56.7%)	224 (55.7%)	232 (57.7%)	0.569
Neurosurgery (%)	572 (25.1%)	479 (25.7%)	93 (22.3%)	0.147	175 (21.8%)	85 (21.1%)	90 (22.4%)	0.669
30-day mortality (%)	404 (17.7%)	371 (19.9%)	33 (7.9%)	**< 0.001**	94 (11.7%)	65 (16.2%)	29 (7.2%)	**< 0.001**
Length of ICU stay (day)	2.3 (1.2–5.6)	2.1 (1.2–5.0)	3.2 (1.7–7.3)	**< 0.001**	2.7 (1.5–6.1)	2.3 (1.3–5.6)	3.0 (1.6–7.1)	**< 0.001**
Length of hospital stay (day)	6.3 (3.6–12.4)	5.9 (3.4–11.5)	9.2 (4.5–17.5)	**< 0.001**	7.2 (3.7–14.8)	5.5 (3.1–11.6)	9.2 (4.5–17.6)	**< 0.001**

GCS, Glasgow Coma Scale; ISS, Injury Severity Score; WBC, white blood cell; RBC, red blood cell. Bold values indicate *p* < 0.05.

### 3.2 Association between the thiamine use and mortality in TBI patients

**Figure 2:**
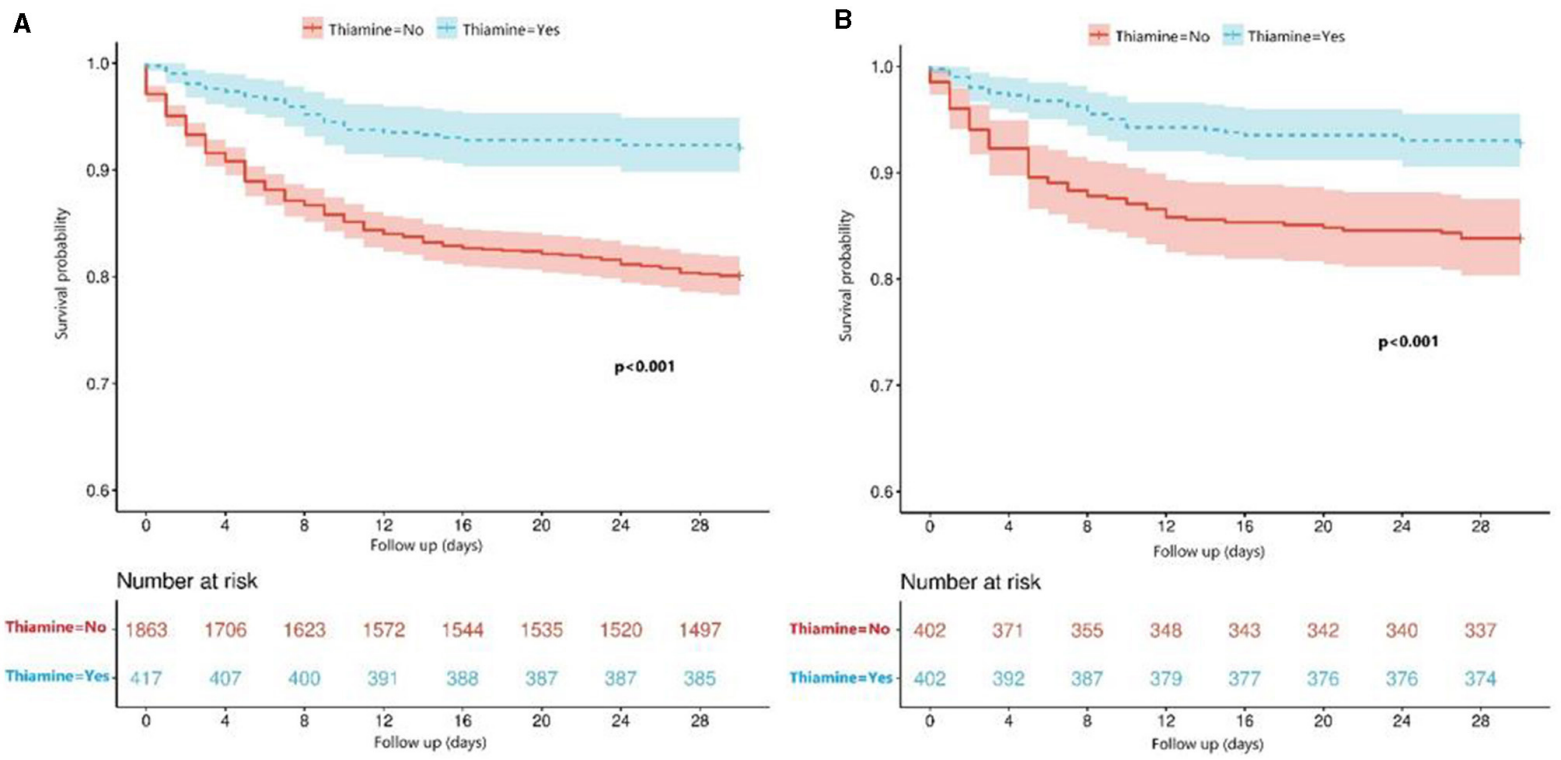
**(A)** Comparison of survival between the thiamine use group and the non-thiamine use group using the Kaplan–Meier survival analysis in the primary cohort before PSM; **(B)** Comparison of survival between the thiamine use group and the non-thiamine use group using the Kaplan–Meier survival analysis in the cohort after PSM.

**Figure 3:**
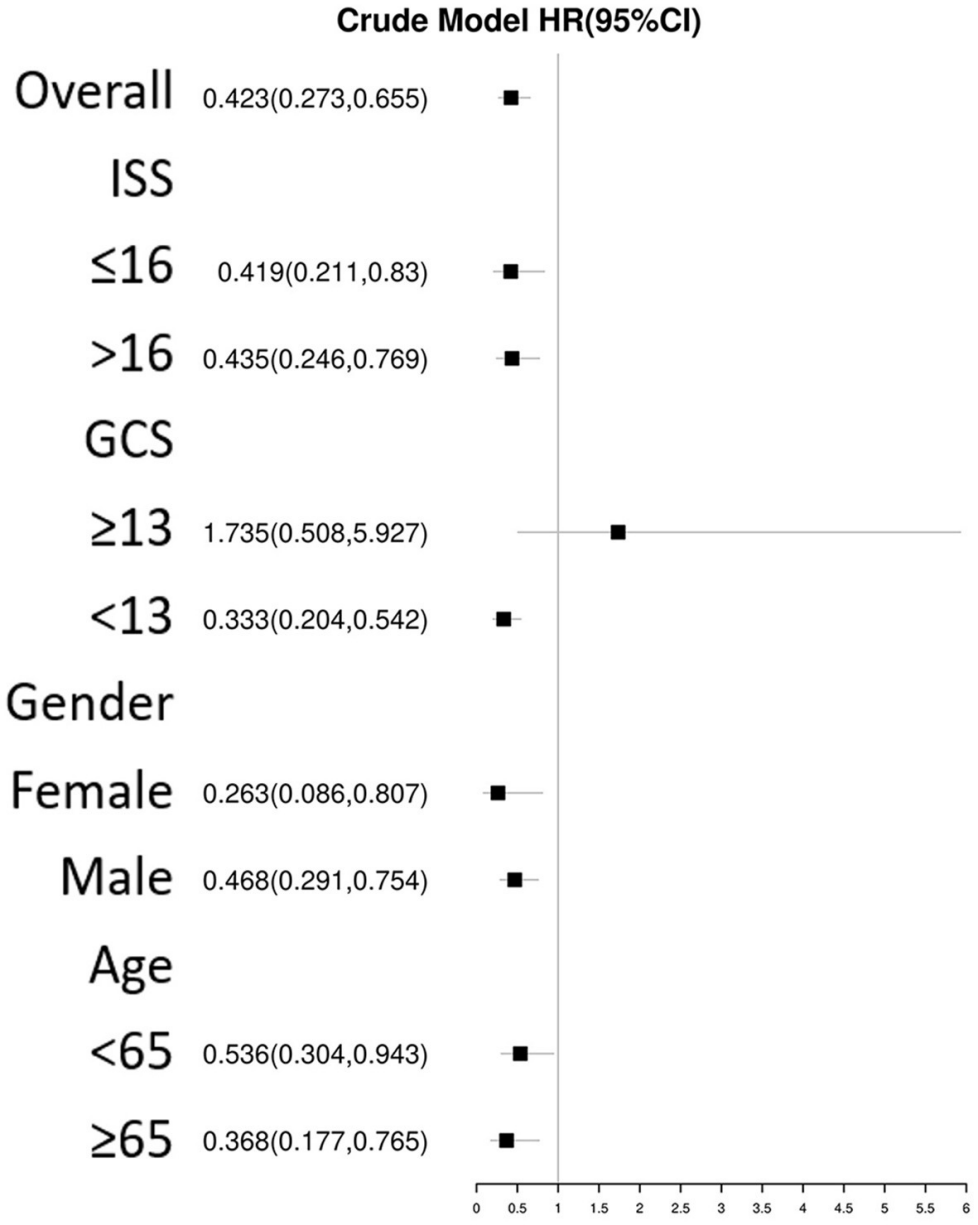
Subgroup analysis of the association between thiamine use and mortality in TBI patients using Cox regression.

**Figure 4:**
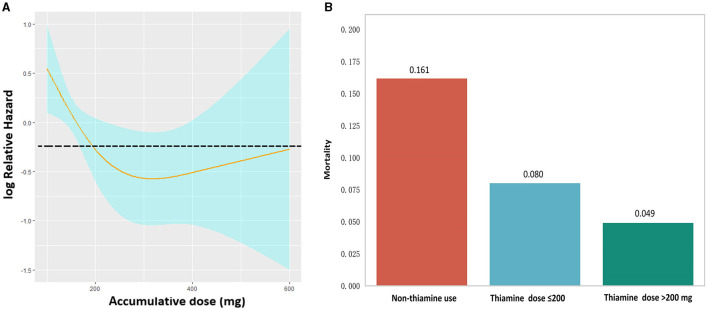
**(A)** Restricted cubic spline showing the association between thiamine dose and mortality in TBI patients. **(B)** Mortality comparison using the Chi-square test between the non-thiamine use group, the thiamine use group with a thiamine dose of ≤ 200 mg, and the thiamine use group with a thiamine dose of >200 mg.

The univariate Cox regression analysis identified several factors significantly associated with mortality in TBI patients. These factors include age (*p* < 0.001), diabetes (*p* = 0.006), chronic renal disease (*p* < 0.001), cancer (*p* = 0.037), diastolic blood pressure (*p* = 0.002), GCS score (*p* < 0.001), ISS score (*p* < 0.001), WBC count (*p* < 0.001), platelet count (*p* < 0.001), hemoglobin level (*p* < 0.001), glucose level (*p* < 0.001), thiamine use (*p* < 0.001), vasopressor during the first day (*p* < 0.001), RBC transfusion during the first day (*p* < 0.001), platelet transfusion during the first day (*p* < 0.001), coagulopathy (*p* < 0.001), mechanical ventilation (*p* < 0.001), and neurosurgery (*p* = 0.001) (Table 2). The multivariate Cox regression analysis confirmed that thiamine use was independently associated with mortality (OR = 0.454, *p* < 0.001) (Table 3). After PSM, thiamine use was still associated with better prognosis, and this association was statistically significant (OR = 0.403, *p* < 0.001). The Kaplan–Meier survival analysis revealed that the thiamine use group had a significantly higher survival rate compared to the non-thiamine use group (*p* < 0.001) (Figures 2A, B). In the sub-group analysis, ISS score, gender, and age did not influence the association between thiamine use and mortality in TBI patients; however, the GCS score did influence the association (Figure 3). Thiamine use is associated with a better prognosis in TBI patients with a GCS score of < 13, indicating moderate to severe TBI, but it is not associated with the prognosis of TBI patients with a GCS score of ≥13, which indicates mild TBI. Finally, the association between thiamine dose and mortality was identified using the RCS curve among TBI patients receiving thiamine in the cohort after PSM (Figure 4A). The cutoff value of the thiamine dose was set at 200 mg. Figure 4B shows different mortality rates between the non-thiamine use group (16.1%), the thiamine use group with a dosage of ≤ 200 mg (8.0%), and the thiamine use group with a dosage of >200 mg (4.9%). The non-thiamine use group had significantly higher mortality rates than both the thiamine use group with a dosage of ≤ 200 mg (< 0.001) and the thiamine use group with a dosage of >200 mg (< 0.001). However, the thiamine use group with a dosage of ≤ 200 mg had higher mortality rates than the thiamine use group with a dosage of >200 mg; however, the difference was not statistically significant (*p* = 0.278).

**Table 2:** Risk factors of 30-day mortality analyzed by univariate Cox regression.

**Variables**	**HR**	**95% CI**	***P*-value**
Age	1.022	(1.017–1.028)	**< 0.001**
Male gender	0.894	(0.733–1.090)	0.268
Diabetes	1.404	(1.102–1.790)	**0.006**
Hypertension	1.105	(0.905–1.349)	0.329
Hyperlipidemia	1.033	(0.777–1.372)	0.824
Coronary heart disease	1.150	(0.871–1.519)	0.324
History of myocardial infarction	0.856	(0.493–1.488)	0.582
Cerebral vascular disease	1.280	(0.661–2.478)	0.464
Liver disease	0.948	(0.575–1.562)	0.833
Chronic renal disease	2.042	(1.519–2.747)	**< 0.001**
Cancer	1.353	(1.018–1.799)	**0.037**
Systolic blood pressure	0.997	(0.993–1.001)	0.110
Diastolic blood pressure	0.990	(0.984–0.996)	**0.002**
Heart rate	0.998	(0.992–1.003)	0.433
GCS	0.830	(0.811–0.849)	**< 0.001**
ISS	1.044	(1.034–1.053)	**< 0.001**
WBC	1.003	(1.001–1.004)	**< 0.001**
Platelet	0.998	(0.997–0.999)	**< 0.001**
Hemoglobin	0.838	(0.803–0.875)	**< 0.001**
Glucose	1.003	(1.003–1.004)	**< 0.001**
Epidural hematoma	1.084	(0.865–1.359)	0.485
Subdural hematoma	1.005	(0.825–1.224)	0.961
Subarachnoid hemorrhage	1.184	(0.974–1.441)	0.090
Intraparenchymal hemorrhage	0.845	(0.653–1.094)	0.201
Thiamine use	0.369	(0.259–0.527)	**< 0.001**
Vasopressor during the first day	3.688	(2.837–4.794)	**< 0.001**
RBC during the first day	2.465	(1.877–3.237)	**< 0.001**
Platelet during the first day	2.022	(1.558–2.624)	**< 0.001**
Coagulopathy	2.213	(1.821–2.690)	**< 0.001**
Mechanical ventilation	4.121	(3.292–5.159)	**< 0.001**
Neurosurgery	1.416	(1.147–1.747)	**0.001**

HR, hazard ratio; CI, confidence interval; GCS, Glasgow Coma Scale; ISS, Injury Severity Score; WBC, white blood cell; RBC, red blood cell. Bold values indicate *p* < 0.05.

**Table 3:** Association between thiamine use and 30-day mortality analyzed by Cox regression.

	**HR**	**95% CI**	***p***
Unadjusted before PSM	0.369	0.259–0.527	< 0.001
Adjusted before PSM	0.454	0.315–0.654	< 0.001
After PSM	0.403	0.254–0.640	< 0.001

HR, hazard ratio; CI, confidence interval; PSM, propensity score match.

## 4 Discussion

This study indicated that thiamine use might lead to an improved prognosis for most TBI patients, including lower 30-day mortality and shorter length of hospital stay. As a water-soluble vitamin essential for cellular metabolism, thiamine exists in several forms in the human body, including thiamine, thiamine monophosphate, and thiamine pyrophosphate (TPP) (25). TPP is closely associated with glycolysis promoting the transformation of glucose to pyruvate and the Krebs cycle, which is essential for ATP production. Therefore, thiamine deficiency could disturb the carbohydrate metabolism with decreased energy production and the accumulation of lactate.

Previous studies have indicated that thiamine deficiency is prevalent among critically ill patients, with an incidence rate of 20% in those admitted to the ICU and 21% in those admitted to the emergency department (26–28). Thiamine deficiency during hospitalizations may be attributable to factors such as increased consumption and inadequate intake of food. Systemic inflammatory response syndrome and oxidative stress commonly occur in conditions like severe sepsis, burns, and trauma, potentially due to the depletion of thiamine storage in the body (29–31). Impaired reabsorption and increased excretion from the kidney due to diuretic use is another important cause of thiamine deficiency (32, 33). Additionally, thiamine deficiency is often observed in alcoholic patients due to malnutrition, inadequate liver storage, and impaired gastrointestinal absorption (34–36). Finally, inadequate intake caused by chronic malnutrition, starvation, parenteral feeding, and chemotherapy could also promote the development of thiamine deficiency (37).

In TBI patients, thiamine deficiency may be caused by diverse factors, including a history of alcoholism, increased consumption due to inflammation and oxidative stress after injury, massive usage of furosemide, and inappropriate parenteral or enteral feeding. Thiamine deficiency could promote the development of lactic acidosis, which has been confirmed to be independently associated with a worse prognosis in TBI patients (38, 39). Furthermore, it can lead to decreased synthesis of TPP, which subsequently impairs the anabolic activity in the brain, including the carbohydrate metabolism, lipid metabolism, and amino acid metabolism. These metabolic activities are essential for the production of ATP, myelin, and neurotransmitters (34). One study involving general ICU patients found that supplementation of antioxidant micronutrients containing zinc, selenium, thiamine, and vitamin C did not reduce the risk of organ failure; however, it alleviated the inflammatory response in patients undergoing cardiac surgery or experiencing trauma (40). Another animal study found that α-ketoglutarate dehydrogenase was the major damaged site in the mitochondria after TBI and was associated with neuroinflammation in the rat model (41). However, thiamine could preserve the mitochondrial function after TBI by preventing the inactivation of α-ketoglutarate dehydrogenase. Therefore, adequate supplementation of thiamine to TBI patients may be essential to alleviate secondary brain injury and improve their prognosis.

Previous database studies have found that thiamine supplementation was associated with reduced mortality rates in various patient groups, including those diagnosed with acute kidney injury, sepsis, or ventilator-associated pneumonia (19–22). Another retrospective matched cohort study confirmed that thiamine supplementation within 24 h of admission was associated with improved lactate clearance and a reduction in 28-day mortality in septic shock patients (42). Because of its water-soluble nature, thiamine can be rapidly recovered in the body through intravenous or oral supplementation. The Recommended Daily Allowance (RDA) suggests the thiamine dose for adults as 1.1–1.2 mg/day, whereas the European Community directive for enteral nutrition recommends a minimum thiamine dose of 1.2 mg/day and a maximum dose of 10 mg/day (43, 44). While these recommendations are tailored for healthy adults, they may not be suitable for critically ill patients with widespread vitamin deficiency and increased vitamin requirements. In our study, the RCS showed an inflection point at the thiamine dose of 200 mg, which indicated that TBI patients receiving accumulative doses of thiamine >200 mg had relatively lower mortality than those without. The statistical significance, generalizability, and reliability of this finding were limited by the sample size and the lack of unadjusted analysis. Additionally, the optimal daily dose of thiamine was not fully explored because it may be influenced by multiple factors such as weight, hepatic and renal function, and baseline nutritional status. The most appropriate dose and the detailed regime of thiamine supplementation for TBI patients deserve further exploration.

This study has several limitations. First, this database study was performed using the dataset from a single medical center. The association between thiamine use and mortality in TBI patients should be further verified in other medical centers through randomized controlled trials. Second, although many clinical factors were matched using the PSM, some potential confounding factors were still not included, which may influence the reliability of the conclusion. Third, the daily dose, initiating time, and duration of thiamine supplementation were not recorded, so we could not explore the optimal regime of thiamine supplementation. Although we have analyzed the relationship between the total dose during hospitalizations and mortality in TBI patients, this relationship may be influenced by many confounding factors such as weight, hepatic and renal function, and baseline nutritional status. Fourth, we only recorded the mortality rate as the primary outcome due to the limited prognostic information in the database. Other outcomes of TBI patients, including functional status and cognitive status, may also be influenced by thiamine use. Finally, the baseline serum level of thiamine was not recorded in the database. Therefore, we could not identify the independent effect of thiamine deficiency or determine which patients would benefit most from thiamine supplementation.

## 5 Conclusion

Thiamine supplementation has been shown to improve survival outcomes in TBI patients, with the exception of those with mild TBI. The optimal daily dose and timing of thiamine supplementation for patients with TBI are worthy to be explored in future studies.

## Data availability statement

The raw data supporting the conclusions of this article will be made available by the authors, without undue reservation.

## Ethics statement

The studies involving humans were approved by the Review board of West China Hospital. The studies were conducted in accordance with the local legislation and institutional requirements. The participants provided their written informed consent to participate in this study.

## Author contributions

RW: Conceptualization, Data curation, Formal analysis, Writing – original draft. YZ: Data curation, Formal analysis, Writing – original draft. MH: Supervision, Validation, Writing – review & editing. JX: Funding acquisition, Project administration, Supervision, Validation, Writing – review & editing.

## References

[1] Dewan MC Rattani A Gupta S Baticulon RE Hung Y-C Punchak M. Estimating the global incidence of traumatic brain injury. JNS. (2019) 130:1080–97. 10.3171/2017.10.JNS17352 29701556

[2] Maas AIR Fitzgerald M Gao G Gupta D Hutchinson P Manley GT. Traumatic brain injury over the past 20 years: research and clinical progress. The Lancet Neurol. (2022) 21:768–70. 10.1016/S1474-4422(22)00307-6 35963251

[3] Zeiler FA Aries M Czosnyka M Smielewski P. Cerebral autoregulation monitoring in traumatic brain injury: an overview of recent advances in personalized medicine. J Neurotrauma. (2022) 39:1477–94. 10.1089/neu.2022.0217 35793108

[4] Scrimgeour AG Condlin ML. Nutritional treatment for traumatic brain injury. J Neurotrauma. (2014) 31:989–99. 10.1089/neu.2013.3234 24605947

[5] Lai JQ Chen XR Lin S Chen CN Zheng XX. Progress in research on the role of clinical nutrition in treating traumatic brain injury affecting the neurovascular unit. Nutr Rev. (2023) 81:1051–62. 10.1093/nutrit/nuac099 36409999

[6] Li Y Liu C Luo X He Q Cheng Y Shen W. Controlling nutritional status score and prognostic nutrition index predict the outcome after severe traumatic brain injury. Nutr Neurosci. (2022) 25:690–7. 10.1080/1028415X.2020.1804097 32778001

[7] Baltazar GA Pate AJ Panigrahi B LaBoy S Prosniak R Mody A. Malnutrition as measured by albumin and prealbumin on admission is associated with poor outcomes after severe traumatic brain injury. Am Surg. (2015) 81:E61–3. 10.1177/000313481508100208 25642858

[8] Krakau K Hansson A Karlsson T de Boussard CN Tengvar C Borg J. Nutritional treatment of patients with severe traumatic brain injury during the first six months after injury. Nutrition. (2007) 23:308–17. 10.1016/j.nut.2007.01.010 17369022

[9] Lucke-Wold BP Logsdon AF Nguyen L Eltanahay A Turner RC Bonasso P. Supplements, nutrition, and alternative therapies for the treatment of traumatic brain injury. Nutr Neurosci. (2018) 21:79–91. 10.1080/1028415X.2016.1236174 27705610 PMC5491366

[10] Lee YH Bang ES Lee JH Lee JD Kang DR Hong J. Serum concentrations of trace elements zinc, copper, selenium, and manganese in critically ill patients. Biol Trace Elem Res. (2019) 188:316–25. 10.1007/s12011-018-1429-4 30047077 PMC6424942

[11] Tan Q Wang Y Zhang G Lu B Wang T Tao T. The metabolic effects of multi-trace elements on parenteral nutrition for critically ill pediatric patients: a randomized controlled trial and metabolomic research. Trans pediatrics. (2021) 10:2579–93. 10.21037/tp-21-456 34765482 PMC8578764

[12] Tang H Hua F Wang J Yousuf S Atif F Sayeed I. Progesterone and vitamin D combination therapy modulates inflammatory response after traumatic brain injury. Brain Injury. (2015) 29:1165–74. 10.3109/02699052.2015.1035330 26083048 PMC4894830

[13] Aiguo W Zhe Y Gomez-Pinilla F. Vitamin E protects against oxidative damage and learning disability after mild traumatic brain injury in rats. Neurorehabil Neural Repair. (2010) 24:290–8. 10.1177/1545968309348318 19841436 PMC2824788

[14] Cope EC Morris DR Scrimgeour AG VanLandingham JW Levenson CW. Zinc supplementation provides behavioral resiliency in a rat model of traumatic brain injury. Physiol Behav. (2011) 104:942–7. 10.1016/j.physbeh.2011.06.007 21699908 PMC3506179

[15] Mills JD Hadley K Bailes JE. Dietary supplementation with the omega-3 fatty acid docosahexaenoic acid in traumatic brain injury. Neurosurgery. (2011) 68:474–81. 10.1227/NEU.0b013e3181ff692b 21135750

[16] Chen G Shi J Qi M Yin H Hang C. Glutamine decreases intestinal nuclear factor kappa B activity and pro-inflammatory cytokine expression after traumatic brain injury in rats. Inf Res Off J Eur Histam Res Soc. (2008) 57:57–64. 10.1007/s00011-007-7101-7 18288455

[17] Depeint F Bruce WR Shangari N Mehta R O'Brien PJ. Mitochondrial function and toxicity: role of the B vitamin family on mitochondrial energy metabolism. Chem Biol Interact. (2006) 163:94–112. 10.1016/j.cbi.2006.04.014 16765926

[18] Bubber P Ke ZJ Gibson GE. Tricarboxylic acid cycle enzymes following thiamine deficiency. Neurochem Int. (2004) 45:1021–8. 10.1016/j.neuint.2004.05.007 15337301

[19] Hu C Wu T Ma S Huang W Xu Q Kashani KB. Association of thiamine use with outcomes in patients with sepsis and alcohol use disorder: an analysis of the MIMIC-III database. Inf Dis Ther. (2022) 11:771–86. 10.1007/s40121-022-00603-1 35169996 PMC8960538

[20] Li X Luan H Zhang H Li C Bu Q Zhou B. Associations between early thiamine administration and clinical outcomes in critically ill patients with acute kidney injury. Br J Nutr. (2022) 128:183–91. 10.1017/S0007114521003111 34392848

[21] Zhang L Li S Lu X Liu Y Ren Y Huang T. Thiamine may be beneficial for patients with ventilator-associated pneumonia in the intensive care unit: a retrospective study based on the MIMIC-IV database. Front Pharmacol. (2022) 13:898566. 10.3389/fphar.2022.898566 35814219 PMC9259950

[22] Baron SW Yu PC Imrey PB Southern WN Deshpande A Rothberg MB. Early treatment with thiamine and mortality among patients with alcohol use disorder who are hospitalized for pneumonia. J Hosp Med. (2022) 17:585–93. 10.1002/jhm.12895 35729853

[23] Dekker SE Duvekot A de Vries HM Geeraedts LM Peerdeman SM de Waard MC. Relationship between tissue perfusion and coagulopathy in traumatic brain injury. The J Surg Res. (2016) 205:147–54. 10.1016/j.jss.2016.06.023 27621012

[24] Alexiou GA Lianos G Fotakopoulos G Michos E Pachatouridis D Voulgaris S. Admission glucose and coagulopathy occurrence in patients with traumatic brain injury. Brain Injury. (2014) 28:438–41. 10.3109/02699052.2014.888769 24564221

[25] Gangolf M Czerniecki J Radermecker M Detry O Nisolle M Jouan C. Thiamine status in humans and content of phosphorylated thiamine derivatives in biopsies and cultured cells. PLoS ONE. (2010) 5:e13616. 10.1371/journal.pone.0013616 21049048 PMC2963613

[26] Lima LF Leite HP Taddei JA. Low blood thiamine concentrations in children upon admission to the intensive care unit: risk factors and prognostic significance. Am J Clin Nutr. (2011) 93:57–61. 10.3945/ajcn.2009.29078 21068344

[27] Berger MM Shenkin A Revelly JP Roberts E Cayeux MC Baines M. Copper, selenium, zinc, and thiamine balances during continuous venovenous hemodiafiltration in critically ill patients. Am J Clin Nutr. (2004) 80:410–6. 10.1093/ajcn/80.2.410 15277163

[28] Cruickshank AM Telfer AB Shenkin A. Thiamine deficiency in the critically ill. Intensive Care Med. (1988) 14:384–7. 10.1007/BF00262893 3136196

[29] Donnino MW Carney E Cocchi MN Barbash I Chase M Joyce N. Thiamine deficiency in critically ill patients with sepsis. J Crit Care. (2010) 25:576–81. 10.1016/j.jcrc.2010.03.003 20646908

[30] Falder S Silla R Phillips M Rea S Gurfinkel R Baur E. Thiamine supplementation increases serum thiamine and reduces pyruvate and lactate levels in burn patients. Burns. (2010) 36:261–9. 10.1016/j.burns.2009.04.012 19501976

[31] Soguel L Chioléro RL Ruffieux C Berger MM. Monitoring the clinical introduction of a glutamine and antioxidant solution in critically ill trauma and burn patients. Nutrition. (2008) 24:1123–32. 10.1016/j.nut.2008.05.024 18692364

[32] Dunn SP Bleske B Dorsch M Macaulay T Van Tassell B Vardeny O. Nutrition and heart failure: impact of drug therapies and management strategies. Nutr Clin Pract. (2009) 24:60–75. 10.1177/0884533608329299 19244150

[33] Sica DA. Loop diuretic therapy, thiamine balance, and heart failure. Congestive Heart Failure. (2007) 13:244–7. 10.1111/j.1527-5299.2007.06260.x 17673878

[34] Zahr NM Kaufman KL Harper CG. Clinical and pathological features of alcohol-related brain damage. Nat Rev Neurol. (2011) 7:284–94. 10.1038/nrneurol.2011.42 21487421 PMC8121189

[35] Galvin R Bråthen G Ivashynka A Hillbom M Tanasescu R Leone MA. guidelines for diagnosis, therapy and prevention of Wernicke encephalopathy. Eur J Neurol. (2010) 17:1408–18. 10.1111/j.1468-1331.2010.03153.x 20642790

[36] Sechi G Serra A. Wernicke's encephalopathy: new clinical settings and recent advances in diagnosis and management. The Lancet Neurol. (2007) 6:442–55. 10.1016/S1474-4422(07)70104-7 17434099

[37] Manzanares W Hardy G. Thiamine supplementation in the critically ill. Curr Opin Clin Nutr Metab Care. (2011) 14:610–7. 10.1097/MCO.0b013e32834b8911 21912244

[38] Fu YQ Bai K Liu CJ. The impact of admission serum lactate on children with moderate to severe traumatic brain injury. PLoS ONE. (2019) 14:e0222591. 10.1371/journal.pone.0222591 31536567 PMC6752785

[39] Svedung Wettervik T Engquist H Howells T Rostami E Hillered L Enblad P. Arterial lactate in traumatic brain injury - Relation to intracranial pressure dynamics, cerebral energy metabolism and clinical outcome. J Crit Care. (2020) 60:218–25. 10.1016/j.jcrc.2020.08.014 32882604

[40] Berger MM Soguel L Shenkin A Revelly JP Pinget C Baines M. Influence of early antioxidant supplements on clinical evolution and organ function in critically ill cardiac surgery, major trauma, and subarachnoid hemorrhage patients. Critical Care. (2008) 12:R101. 10.1186/cc6981 18687132 PMC2575590

[41] Mkrtchyan GV Üçal M Müllebner A Dumitrescu S Kames M Moldzio R. Thiamine preserves mitochondrial function in a rat model of traumatic brain injury, preventing inactivation of the 2-oxoglutarate dehydrogenase complex. Biochimica et Biophys Bioenergetics. (2018) 1859:925–31. 10.1016/j.bbabio.2018.05.005 29777685

[42] Woolum JA Abner EL Kelly A Thompson Bastin ML Morris PE Flannery AH. Effect of thiamine administration on lactate clearance and mortality in patients with septic shock. Crit Care Med. (2018) 46:1747–52. 10.1097/CCM.0000000000003311 30028362

[43] Shenkin A. Basics in clinical nutrition: Trace elements and vitamins in parenteral and enteral nutrition. E Spen Eur E J Clin Nutr Metab. (2008) 3:e293–e7. 10.1016/j.eclnm.2008.07.011

[44] Sriram K Lonchyna VA. Micronutrient supplementation in adult nutrition therapy: practical considerations. JPEN. (2009) 33:548–62. 10.1177/0148607108328470 19454751

